# Fear learning through the two visual systems, a commentary on: “A parvalbumin-positive excitatory visual pathway to trigger fear responses in mice”

**DOI:** 10.3389/fncir.2015.00056

**Published:** 2015-10-02

**Authors:** Ti-Fei Yuan, Huanxing Su

**Affiliations:** ^1^School of Psychology, Nanjing Normal UniversityNanjing, China; ^2^State Key Laboratory of Quality Research in Chinese Medicine, Institute of Chinese Medical Sciences, University of MacauMacao, China

**Keywords:** fear learning, two visual system, Amygdala, superior colliculus, optogenetics, dopamine, visual cortex, visual thalamus

The theory of two visual systems was firstly proposed in 1960s, to explain the distinct neural mechanisms underlying visual discrimination and localization in rodents and frogs. A recent study demonstrated that fear learning could be transmitted either through visual cortex or simply superior colliculus to the amygdala.

Early brain lesion studies found that tectal lesion is associated with the dysfunction of object localization and the visual cortical lesion is associated with failure in pattern discrimination (Schneider, 1969; Ingle, 1973). Similar idea was proposed for human visual system as well: the two streams hypothesis (Botez, 1975; Milner and Goodale, 2008). It is believed that the dorsal visual system receives whole retinal inputs in fast transmission manner, contributing to visually guided behaviors; while the ventral visual system creates imagery with spatial details, and is highly relevant to visual consciousness.

The traditional view suggested that fear cues are evaluated by visual cortex and then transmitted to the amygdala, through the visual thalamus. However, it is then realized that healthy human subjects could detect “unseen” fearful cues through subcortical connections between right amygdala, pulvinar, and superior colliculus (SC) (Morris et al., 1999; Tamietto and de Gelder, 2010). This highlighted the possibility of “non-conscious” or cortex-independent processing of visual fear cues. The hypothesis is further proved on human patients with cortical blindness, which showed intact fear learning to the visual cue (Hamm et al., 2003). These evidences argued for the presence of subcortical pathway in visual cue dependent- fear learning. Lesions of lateral geniculate body (LG) and lateral posterior nucleus (LP, pulvinar like structure in rodent) of the thalamus together, but not respectively, led to impairment of visual cue-paired fear conditioning (Shi and Davis, 2001). Yet, the exact neural circuits underlying the visual fear guided behavior was unknown. A recent study employed optogenetic dissection of such circuits to answer the question.

One previous study investigated the innate defensive response of mice to overhead looming stimuli as the fear behavior paradigm (Wei et al., 2015). When the authors silenced a group of excitatory neurons by expression and following light activation of NpHR in the superficial layer of SC, the innate fear behavior is eliminated. The authors then injected retrograde trans-synaptic tracers into the amygdala, and identified LPN as the relay in SC-amygdala pathway. Indeed, activating SC-expressed ChR2 axon terminals in LPN was sufficient to elicit the freezing behavior. Collectively, their results suggested that the excitatory pathway from SC to LPN, and then the LPN to amygdala pathway is critical for the amygdala expression of this visual fear cue guided behavior. It is still unknown if the LPN-amygdala pathway is excitatory or inhibitory.

Interestingly, Wei et al. found that Parvalbumin (PV) positive neurons (classical cortical fast-spiking interneurons) in SC are also transfected by CaMKII-promoter virus. In the recent study, Shang et al. further examined these PV neurons and found that SC PV neurons have distinct properties to cortical PV neurons in terms of morphology, electrophysiological characteristics, and biochemical content (Shang et al., 2015). These SC PV neurons are found to be excitatory, and the optogenetic activation of these neurons raised fear response behaviors. Anatomical tracing study confirmed that these SC PV neurons project to parabigeminal nucleus (PBGN), which then project to amygdala. Optogenetic activation of axon terminals from SC PV neuron within PBGN could generate the fear response, confirming that the PV+ SC-PBGN-Amygdala is a non-canonical fear pathway to visual cues. It is suggested that the expression of Parvalbumin is critical to drive the fast response required for this pathway (Figure 1).

**Figure 1:**
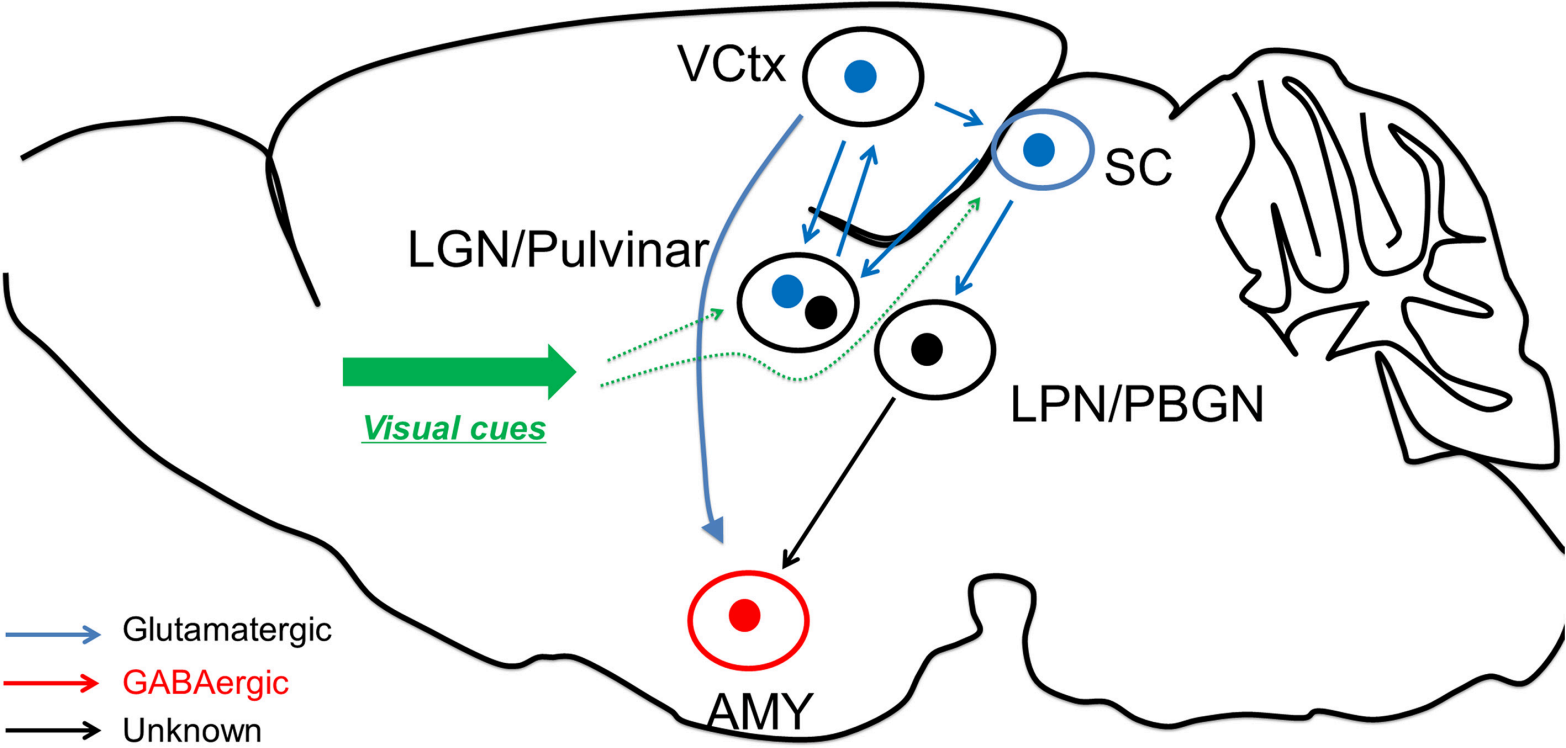
**Fear learning through the two visual systems**. The visual discrimination system receives information from visual thalamus and transmits the information to visual cortex for fear detection, before reaching amygdala. On the other hand, the superior colliculus could directly excite amygdala neurons through LPN or BPGN pathway, potentially in an “unconscious” manner.

The results of Shang et al. may speak to the long debate on the visual mechanism underlying fear conditioning. In particular, is visual cortex required for the fear response behavior to visual cues? The brain lesion study or physiological recordings showing that SC neurons respond to fear behavior could be explained by compensation mechanism or reciprocal neural circuits between visual cortex and the SC or SC-projecting brain regions. For a stronger test of the hypothesis, future studies may wish to combine optogenetic excitation of SC pathway and optogenetic silencing of the visual cortex and LGN pathway, or with muscimol infusion into the visual cortex to exclude the collateral excitation or reciprocal neural circuits involved in fear responses described above.

Knowing that the fear learning can be mediated by the SC-LPN/PBGN-Amygdala pathway raises a new set of questions. How does the pathway discriminate fearful or neutral information? Are there any special retina ganglion cells responsible for the fear response behavior? What other pathways may brain employ in parallel? For instance, direct retinal projection to dorsal raphe is known to regulate the affective behavior (Ren et al., 2013). We now have the optogenetic tools to answer these possibilities, and to answer the psychological theory “two visual systems” from the fear learning perspective.

## Conflict of interest statement

The authors declare that the research was conducted in the absence of any commercial or financial relationships that could be construed as a potential conflict of interest.
